# Case Report: Complete pathologic response in advanced melanoma with SFRT and dual checkpoint inhibition

**DOI:** 10.3389/fonc.2025.1697902

**Published:** 2025-10-17

**Authors:** Jared Hobson, Michael P. Grams, Joanina K. Gicobi, Dennis Wigle, Lisa A. Kottschade, Lindsey A. Durham, Kimberly Corbin, Haidong Dong, Svetomir N. Markovic, Sean S. Park

**Affiliations:** ^1^ Department of Radiation Oncology, Mayo Clinic, Rochester, MN, United States; ^2^ Department of Medical Oncology, Mayo Clinic, Rochester, MN, United States; ^3^ Department of Thoracic Surgery, Mayo Clinic, Rochester, MN, United States; ^4^ Department of Immunology, Mayo Clinic, Rochester, MN, United States

**Keywords:** case report, melanoma, SFRT, radiation, immunotherapy

## Abstract

Immune checkpoint inhibitors (ICIs) have transformed the treatment landscape for advanced melanoma, though response rates remain limited in bulky disease. Herein, we report the case of a complete pathologic response following combination spatially fractionated radiation therapy (SFRT) and dual nivolumab and ipilimumab for a 12 cm right lung melanoma mass, with subsequent lobectomy revealing no viable tumor cells. Now disease free 2.5 years after treatment, including more than 1 year off all systemic therapy, this case highlights the potential synergy between SFRT and immunotherapy in advanced melanoma management.

## Introduction

The treatment landscape for melanoma has evolved significantly with the advent of immune checkpoint inhibitors (ICIs), transforming outcomes in advanced stage and metastatic disease. While long term results from CheckMate 067 demonstrated a durable response with dual nivolumab and ipilimumab, objective response rates were 58% for combination therapy, with approximately 40% of patients having a minimal response to ICI (1–3). As such, various patient and tumor characteristics have been explored to predict treatment response. Tumor volume has been negatively correlated with ICI efficacy, with large tumors demonstrating immune exclusion, hypoxia, and poor antigen presentation, limiting treatment efficacy (3, 4). Radiation therapy may be an optimal tool in this setting, recognized for its ability to reduce tumor burden and increasingly for immunomodulatory properties that may augment systemic immune response (5).

Historically, melanoma has been considered radioresistant, and while high dose radiotherapy with stereotactic body radiation therapy (SBRT) has shown promise, bulky tumors tend to exhibit worse local control and are often limited by nearby organs at risk (OARs) (6). As such, ASTRO guidelines conditionally recommend SBRT for tumors >5cm (7). Spatially fractionated radiation therapy (SFRT) offers a novel alternative in such cases, creating a heterogeneous dose distribution of high and low-dose regions within a tumor while respecting OAR constraints. This technique has demonstrated enhanced therapeutic outcomes with reports of significant symptom relief and greater-than-expected tumor responses (8–10). While the mechanisms for this are poorly understood, SFRT has been shown to modulate the tumor microenvironment and immune response, leading to bystander and abscopal effects (11–13). Given its potential synergy with immunotherapy, increasing interest has grown in coupling SFRT with ICI for bulky tumors (14).

Here, we present the case of a bulky metastatic melanoma mass involving the right lung treated with SFRT and concurrent nivolumab and ipilimumab, leading to a complete pathologic response within 5 months of treatment with low toxicity. Currently disease free 2.5 years after treatment and off all systemic therapy for over one year, this case highlights the potential for SFRT to enhance ICI efficacy and underscores the role of multimodal therapy in melanoma management.

## Case presentation

**Figure 1:**
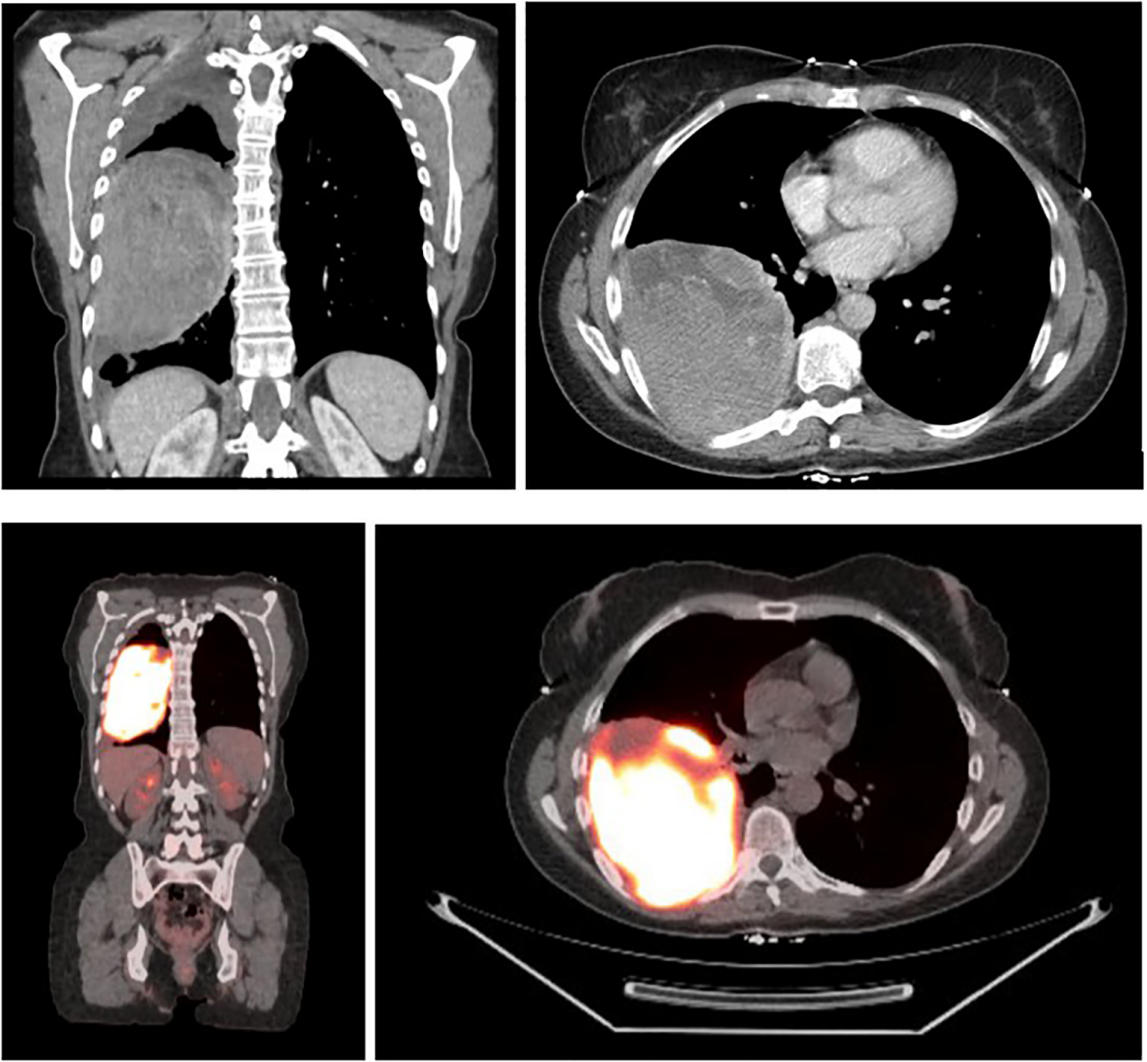
CT chest 5/25/2022 demonstrating a 12.0 x 9.1 cm right lower lobe mass, with FDG F-18 PET 6/3/2022 noting avidity and no evidence of metastatic disease, SUV max 24.6.

A 58-year-old female with a history of occasional social smoking (quit in 1993) and non-melanomatous skin cancer presented with four months of progressive dyspnea and cough in May 2022. Chest x-ray showed a large right-sided mass, with Computed Tomography (CT) Chest 5/2022 confirming a 12.0 x 9.1 cm heterogeneously enhancing right lower lobe mass abutting the right major fissure and extending into the right middle and upper lobes (**Figure 1**). Small indeterminate pulmonary nodules were seen, with a small right pleural effusion and no significant adenopathy. Subsequent FDG PET and brain MRI revealed an intensely avid primary thoracic mass with no evidence of metastatic disease. Outside bronchoscopy with biopsy was suggestive of melanoma, with negative lymph node stations 7, 10R, and 11L. Repeat CT-guided biopsy 6/2022 confirmed malignant melanoma, BRAF wild-type, with immunohistochemistry positive for SOX10, S100, HMB45, Melan A, and H3K27me3. Clinical exam revealed no evidence of a primary lesion, and differentials included primary pulmonary melanoma versus metastatic melanoma with an unknown primary, the latter favored due to the rarity of primary pulmonary disease. The patient denied any history of melanoma, but did note a family history of melanoma in her father and sister, along with other malignancies, including lymphoma, breast, prostate, and pancreatic cancer. Guardant 360 testing was performed showing somatic alterations in TP53 (5.7% cfDNA), GNAS (0.3%), and APC (1.1%), with no MSI-High detected.

**Figure 2:**
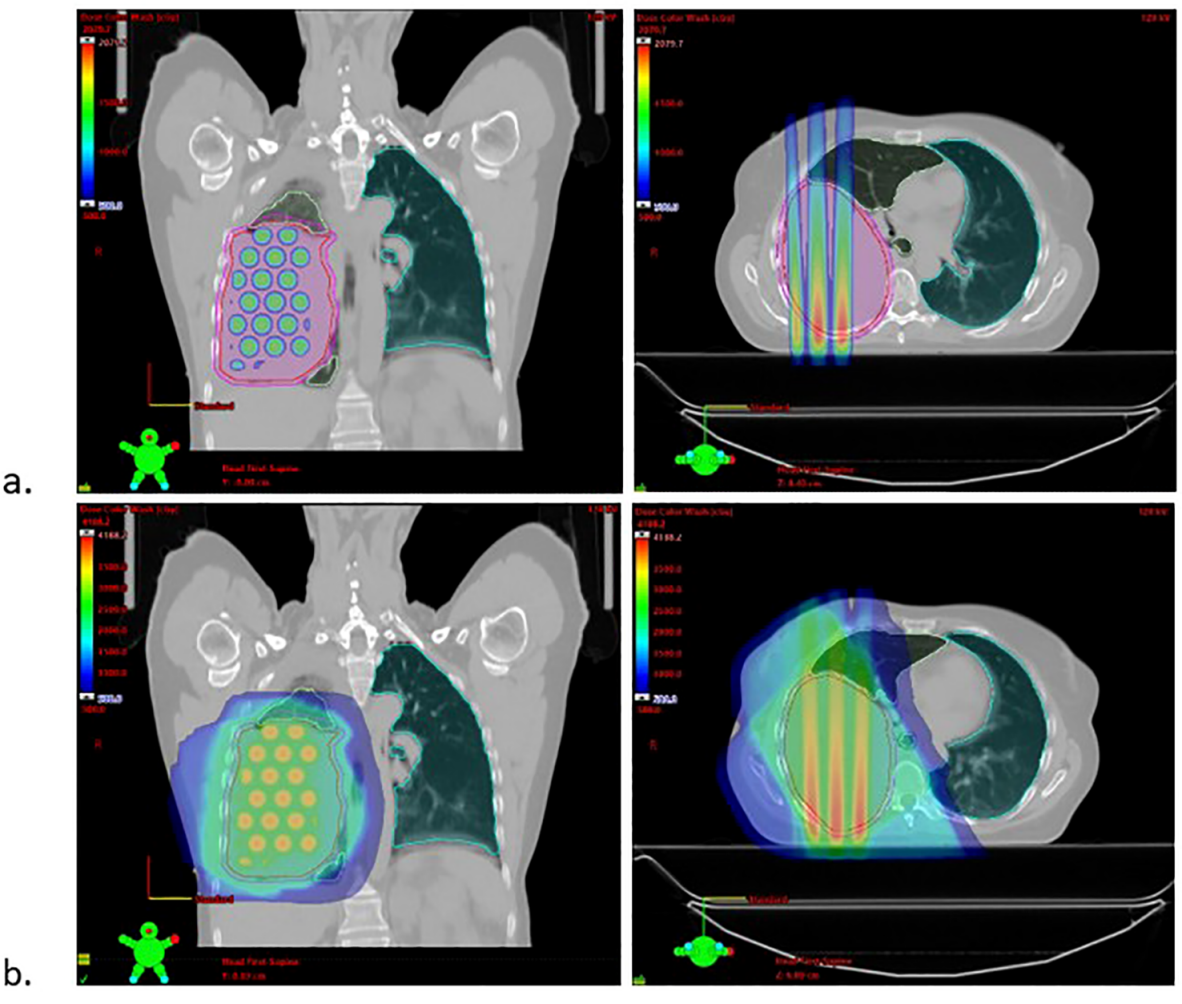
**(a)** Radiation Plan: SFRT plan 20 Gy in 1 fraction **(b)** Sum plan including 20 Gy in 4 fractions. D0.03 of 41 Gy.

Initial discussion with medical oncology, pulmonology, and thoracic surgery recommended neoadjuvant immunotherapy followed by surgical resection. Given the tumor bulk, radiation oncology was consulted with the aim of shrinking the mass, bolstering immune response, and potentially increasing the efficacy of systemic therapy. To this end, the patient was enrolled on clinical trial ROR1903 and treated with Brass GRID SFRT 20 Gy in 1 fraction on 7/1/2022, followed by 20 Gy in 4 fractions between 7/5-7/8/2022 (**Figure 2**). Concurrent nivolumab (1 mg/kg) and ipilimumab (3 mg/kg) were administered every 21 days with first administration 2 days prior to SFRT on 6/29/2022.

**Figure 3:**
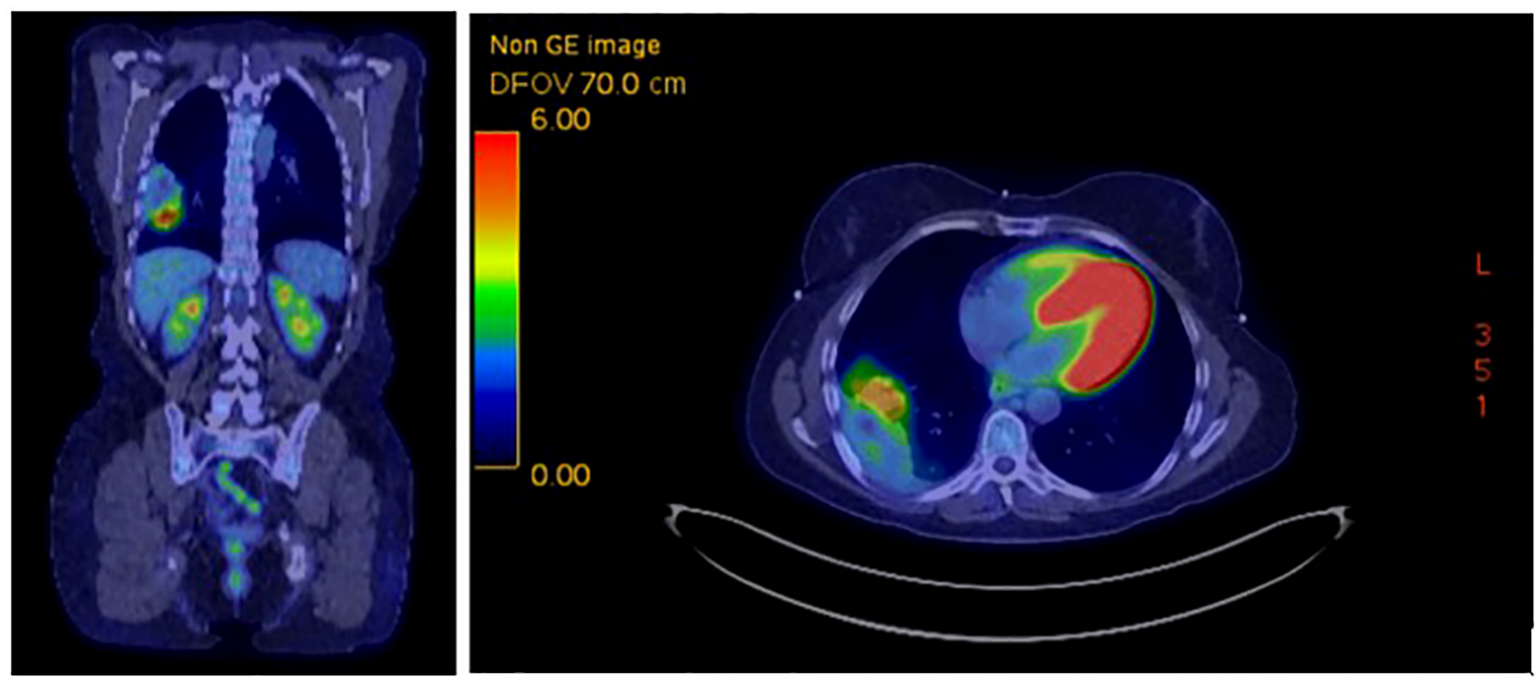
FDG PET 9/22/2022 showing marked response of right lower lobe mass, 6.1 x 3.6 cm with SUV max 6.1.

Radiation was well tolerated, and she continued dual immunotherapy until August 2022 at which time she developed grade 3 hepatitis and a grade 2 rash, necessitating a treatment hold and initiation of high-dose prednisone. At that time, CT imaging showed significant tumor shrinkage down to 7.5 x 3.5 cm with no new evidence of disease. Given her immune-related toxicity, ipilimumab was discontinued, and she was rechallenged with nivolumab monotherapy every two weeks in October 2022 and continued low dose prednisone. Interval PET imaging 9/2022 noted marked response to therapy, with the mass measuring 6.1 x 3.6 cm with an SUV max of 6.1, decreased from 18.7 previously (**Figure 3**). New peripheral consolidative and ground glass infiltrates concerning for radiation pneumonitis were identified, requiring prolonged corticosteroid tapering.

**Figure 4:**
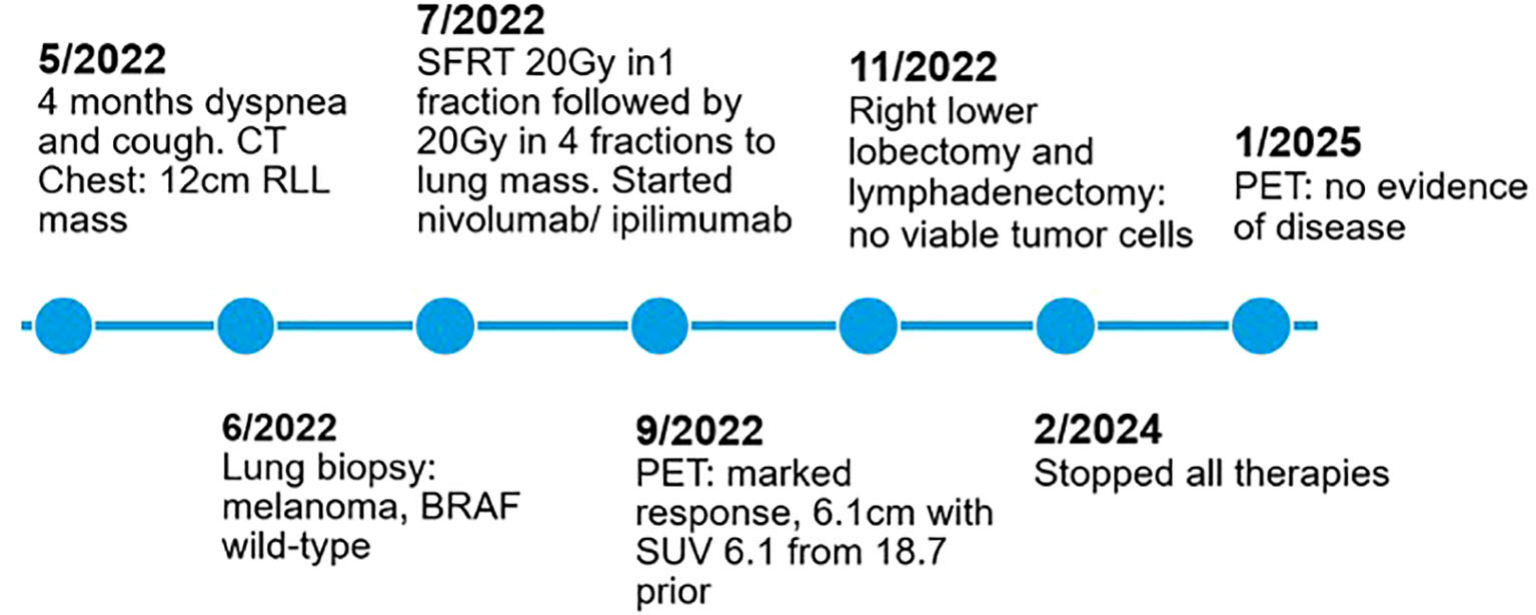
Timeline.

Given persistent PET-avidity, she was evaluated for surgical resection. PFTs showed FEV1 90% predicted and DLCO 78% predicted, and in November 2022, she underwent a right lower lobectomy and mediastinal lymphadenectomy. Final pathology revealed a necrotic 4.1 cm mass with no viable tumor and all lymph nodes negative (0/7), confirming a complete pathologic response. Postoperatively, she resumed adjuvant nivolumab in January 2023 with serial imaging continuing to show no evidence of disease. Given her excellent response, she stopped all therapy in February 2024 and remains disease free to date, with most recent PET 1/2025 continuing to demonstrate no evidence of disease. A summary timeline of key events and treatments can be seen below in **Figure 4**.

## Discussion

Radiation has increasingly been recognized for immunomodulatory effects, both suppressive and stimulatory depending on dosage and fractionation (14, 15). Interest has grown in harnessing these effects with immunotherapy, and this case adds evidence to support the safety and efficacy of SFRT and dual ICIs in treating bulky disease – in this instance providing a complete pathologic response.

### Immunotherapy in melanoma

While immunotherapy use in melanoma dates back to 1984 with IL-2, effects were mixed and toxicity was significant (16). Immune checkpoint inhibitors (ICI) redefined the treatment of advanced melanoma in 2011 with the approval of ipilimumab, a CTLA-4 inhibitor, following the results of MDX010–20 showing significant improvement in survival with acceptable toxicity (17, 18). This was rapidly followed by PD-1 inhibitors Pembrolizumab (KEYNOTE-001, -002) and Nivolumab (CheckMate 037) showing benefit over standard-of-care chemotherapy (19). CheckMate 067 formed a landmark trial in 2015, establishing the benefit of combined nivolumab and ipilimumab, with long term results showing a median overall survival of 72 months, improved from less than 12 months before ipilimumab in 2011, and a melanoma specific survival of 55% at 7.5 years. Notably, for patients who were progression free at 3 years, 10 year melanoma specific survival was 96% (1).

Despite these advances, a significant subset of patients exhibit primary or acquired resistance, with approximately 40% having no response to therapy and another 30-40% experiencing an initial response with subsequent progression (20). While the mechanisms of resistance remain poorly understood, they include defective antigen presentation, immunosuppressive signaling, alternative immune check point activation, and a non-inflamed or “cold” tumor microenvironment (TME) (20–22).

Since ICI efficacy largely relies on amplifying pre-existing anti-tumor T cell responses, the TME and distribution of immune cells within a tumor play a critical role in ICI response. Broadly, tumors consist of tumor parenchyma, with nests of tumor cells, and tumor stroma containing blood vessels, connective tissue, and inflammatory cells (23). Distinct immune profiles have been described relating to this, including immune-active or “hot” tumor phenotypes characterized by parenchymal lymphocytic infiltration, and immune-desert or “cold” phenotypes lacking lymphocytic infiltration. Immune exclusion represents a third phenotype, in which immune cells are abundant within the TME but fail to penetrate the tumor parenchyma (23). Large tumors exhibit increased heterogeneity and immune exclusion, and tumor burden has been negatively correlated with ICI response (3, 24). Additionally, bulky masses harbor a greater proportion of immunosuppressive cells and cytokines, dampening both local and systemic immune responses (24). As such, additional strategies to overcome resistance and enhance response rates are needed, particularly in bulky disease, with radiation therapy offering promise as a synergistic modality.

### Radiation immunomodulation

In addition to causing tumor cell death via direct DNA damage and free radical (ROS) generation, radiation may induce a series of biologic and immune-mediated effects locally and systemically. Local bystander effects, where non-irradiated neighboring cells respond through cell signaling, and distant abscopal effects, where tumor lesions outside the radiation field shrink or disappear, have been well documented in the literature (5). Data evaluating the mechanisms behind these effects have revealed complex immunosuppressive and immunostimulatory properties of radiation.

Beyond its direct cytotoxic effects, radiation may induce immunogenic cell death, releasing neoantigens and damage-associated molecular patterns (DAMPS). These molecules activate antigen-presenting cells, particularly dendritic cells, which in turn prime cytotoxic T cells and bolster immune response (5, 14). Additionally, radiation has been shown to increase the release of pro-inflammatory cytokines and chemokines, upregulate programmed death ligand 1 (PD-L1) expression, and enhance major histocompatibility class I (MHC-1) surface expression (15, 25). The cGAS-STING pathway may also be activated, further promoting immune cell maturation, activation, and polarization, and remodeling the TME (25). These effects may increase lymphocytic infiltration, converting a cold tumor into a hot tumor and overcoming immune exclusion, thereby improving ICI efficacy (26).

Immunosuppressive effects may also arise however, predominantly mediated by the recruitment of myeloid-derived suppressor cells (MDSCs) and regulatory T cells (Tregs), along with the release of immunosuppressive cytokines (27). Additionally, radiation-induced changes in tumor vasculature may exacerbate hypoxia, hinder drug distribution, and limit lymphocyte infiltration. The balance between these contrasting effects appears heavily dependent on dose and fractionation. While conventional fractionation tends to be immunosuppressive, higher doses per fraction may trigger both stimulatory and suppressive responses. Hypofractionated regimens for example, are associated with a greater type-1 interferon (IFN-I) response, while ablative doses, such as 20 Gy in 1 fraction, can induce extensive cell death and release of cytosolic DNA, deplete radioresistant suppressive immune cells, and enhance CD8+ lymphocyte-mediated antitumor activity (28). These effects appear transient however, as subsequent recruitment of MDSCs and Tregs can induce an immunosuppressive environment, limiting the duration of the antitumor response (28, 29). Additionally, ablative doses may significantly damage endothelial cells and disrupt vasculature.

In contrast, low-dose radiotherapy induces less DNA damage, but may reshape the TME to foster a favorable immune response, enhancing both innate and adaptive immunity. It may polarize macrophages toward a M1 phenotype, increasing TNF alpha and IL-12 production and supporting immune recognition. Unlike high dose radiation, it may also promote normalization of the tumor vasculature, improving oxygenation and immune cell infiltration, thereby enhancing ICI response (29). These immunologic effects of low dose radiation alone may not be significant enough to overcome tumor growth however, particularly in the case of bulky disease or radioresistant histologies such as melanoma.

With a spectrum of immunomodulatory effects and distinct dose-response profiles, the optimal dose and fractionation for radiation use with immunotherapy has yet to be defined and may vary between histologies. Pre-clinical data evaluating radiation with anti-CTLA-4 antibodies has suggested that fractionated regimens may elicit a greater abscopal effect than single fraction (30). Additionally, combined regimens utilizing both high and low-dose radiation may harness the immunologic properties of both to achieve synergistic results (31). By creating a heterogenous dose distribution, the enhanced therapeutic ratio and greater-than-expected responses seen with SFRT likely hinge upon this effect and may be ideal for use with ICIs in bulky disease. Additionally, administering a conventional radiation treatment after SFRT, such as the 20 Gy in 4 fractions this patient received, appears to improve response rates, possibly reflecting the impact of fractionation and varying dose responses.

### Spatially fractionated radiation therapy and immunotherapy

Dating back to 1909, SFRT was originally designed as a method of delivering high doses of radiation to a tumor while avoiding detrimental toxicity, particularly to the skin and subcutaneous tissue. By delivering high dose radiation through a physical block with holes called a GRID, a non-uniform dose distribution was created, described as having high dose “peaks” and low dose “valleys” (32). The interspersing of low dose regions between the high dose radiation allowed for greater normal tissue recovery and increased OAR tolerance. Cases utilizing this technique have reported significant symptom relief and tumor shrinkage, thought to be immunologic in nature with bystander and abscopal effects. Modern technology and image guidance have expanded use of SFRT with 3D and VMAT planning, improving targeting and safe delivery (33). This has allowed treatment of tumors located throughout the body, and expanded SFRT techniques. In addition to traditional 2D GRID, 3D LATTICE, microbeam, and minibeam techniques have been developed, altering beam thickness and spatial distribution in hopes of optimizing immunologic outcomes and applicability across tumor sizes and locations (14). SBRT-based PArtial Tumor irradiation of HYpoxic clonogenic cells (SBRT-PATHY), represents another form of SFRT specifically targeting hypoxic regions in bulky masses to potentiate response. Excellent clinical outcomes and abscopal effects have been reported and investigation into the utility and selection for SFRT remain ongoing (34).

Regarding the use of radiation with immunotherapy, responses vary significantly between studies, reflecting differences in histology, molecular alterations, radiation dose and fractionation, chemotherapy and immunotherapy agents, and unique individual biology. Several conventionally fractionated trials such as the PACIFIC trial and CheckMate 577 have demonstrated benefit to adjuvant ICI with definitive radiation (27). Conversely, conflicting results have been seen between studies such as KEYNOTE-A18 and CALLA, and other trials such as the JAVELIN trial have failed to show significant improvement (27, 35, 36). In the high-dose setting, SBRT to a single tumor site followed by pembrolizumab has shown increased overall response rates in advanced non-small cell lung cancer (37). A prospective study combining hypofractionated radiation in metastatic cancers, including melanoma, with PD-1 inhibitors also demonstrated prolonged and complete responses, including patients who had previously progressed while on PD-1 inhibitors (38). Data has been mixed however, with a randomized phase I/II trial of pembrolizumab with SBRT or hypofractionated radiation showing no benefit in progression free or overall survival compared to pembrolizumab alone (39). Further data is needed to full understand the impact of radiation, including dose and fractionation, tumor size and histology, and immunotherapy timing.

While no large trials exist utilizing SFRT with immunotherapy, case reports such as this support concurrent use and suggest synergistic results. Jiang et al. reported a case of LATTICE SFRT combined with anti-PD1 immunotherapy in a patient with metastatic non-small cell lung cancer who achieved a complete response 5 months following concurrent treatment (40). Likewise, Mohiuddin et al. reported a case of advanced melanoma with acquired resistance to multiple systemic agents, including ipilimumab, IL-2, and pembrolizumab, that subsequently had a complete response following GRID SFRT and pembrolizumab, suggesting combination therapy may re-sensitive patients to treatment (41). This case represents another such example, demonstrating a complete pathologic response with minimal radiation induced toxicity aside from pneumonitis treated with corticosteroids. Now almost a few years post treatment, including a year off any therapy, her case supports the use of SFRT with ICIs, offering hope for those with bulky disease.

### Limitations and future directions

While this case highlights a successful treatment using SFRT and immunotherapy, the degree to which each component contributed to her response remains unknown. Robust responses to immunotherapy alone have been seen in melanoma, and the degree to which radiation altered her outcome cannot be ascertained (3). Furthermore, while bulky tumors may have a reduced response to ICI therapy, it remains unclear if dual ICI therapy may overcome this. Nevertheless, the immunomodulatory effects of radiation and potential to overcome barriers such as hypoxia suggest synergistic potential. Continued studies evaluating the combined effects of SFRT and immunotherapy may help elucidate the impact of multimodal therapy and refine treatment regimens.

## Conclusion

While immunotherapy has transformed outcomes in advanced melanoma, responses rates are limited, particularly in bulky disease. This case highlights the potential for SFRT to enhance ICI efficacy in advanced melanoma, leading to a complete pathologic response with minimal radiation-related toxicity. While the contribution of each treatment modality remains uncertain, the significant and sustained tumor remission suggests a synergistic effect between SFRT and dual ICI therapy. Given the challenges of treating bulky melanoma and the limitations of immunotherapy in this setting, SFRT represents a promising strategy to reshape the tumor microenvironment, improve immune infiltration, and augment systemic response. This case adds to the growing evidence supporting the integration of SFRT with immunotherapy and underscores the potential for multimodal approaches to improve outcomes in advanced melanoma. Future studies are needed to optimize radiation dose, fractionation, and patient selection to maximize outcomes.

## Data Availability

The original contributions presented in the study are included in the article/supplementary material. Further inquiries can be directed to the corresponding authors.
